# Planned Tunnel Convergence for Concomitant Posterior Cruciate Ligament Reconstruction and Meniscal Root Repair

**DOI:** 10.1016/j.eats.2025.103633

**Published:** 2025-05-20

**Authors:** Nicholas Newcomb, Ryan Price, Kathryn Yeager, Colin Carroll, Joseph Esquibel, Dustin Richter, Christopher Shultz, Gehron Treme

**Affiliations:** Department of Orthopaedics and Rehabilitation, University of New Mexico, Albuquerque, New Mexico, U.S.A.

## Abstract

Meniscal root injuries frequently occur in the setting of multiligamentous knee injuries. These present a complicating factor for planned tunnels and fixation owing to their proximity to the posterior cruciate ligament (PCL) tibial tunnel. Techniques typically aim to avoid tunnel convergence to reduce the risk of damage to grafts and/or fixation devices and decrease the risk of osteolysis or loss of fixation. We describe a method for planned tunnel convergence for PCL reconstruction and meniscal root repair. The PCL tibial tunnel is first drilled in the typical trajectory. An anterior cruciate ligament drill guide is then placed at the appropriate footprint for the medial or lateral meniscal root, with the starting point at the proximal tibia sharing the same external tibial tunnel aperture as the PCL. Sutures capturing the meniscal root and the PCL graft can then both be passed and exit distally via the same converging tunnel and can be fixed to the proximal tibia using standard techniques. The described technique can be used for either the medial or lateral meniscal root with concomitant PCL reconstruction in a safe and controlled method to reconstruct the native anatomy and avoid tunnel collision with inadvertent injury to grafts or fixation.

Multiligamentous knee injury (MLKI) presents a complex spectrum of pathology that requires careful preoperative planning in terms of management of concomitant injuries, selection and preparation of grafts, order of reconstruction, tunnel position, and methods of fixation. Tunnel convergence is a concern given the multiple structures requiring repair and/or reconstruction and the limited available bony footprint to optimize fixation. Unintended tunnel collision can lead to damage to the graft or can compromise fixation.

Meniscal root injuries occur in the setting of cruciate ligament injuries at a rate of 2% to 10%.^1^^,^^2^ Restoration of the meniscal root is critical for restoring knee biomechanics.^3^ Combined posterior cruciate ligament (PCL) and meniscal root injuries present a risk of tunnel collision owing to the proximity of the root to the tibial tunnel.^2^^,^^4^ Several techniques have been described for tunnel management in these injuries, including using the same tunnel for passage of both the meniscal repair suture and PCL graft or reorienting the tunnels in the sagittal plane to be parallel.^5^^,^^6^ However, these techniques have their own limitations.

This article describes a technique for planned convergence of tunnels for reconstruction of the PCL and repair of the medial or lateral meniscal root. The same tibial aperture is used to drill the meniscal root tunnel from within the PCL tibial tunnel and diverge to the appropriate intra-articular footprint. This allows for a controlled tunnel collision and avoids compromise to both the PCL graft(s) and root repair. Indications are described in Table 1.

**Table 1:** Indications

Acute grade 3 PCL rupture with concomitant lateral or medial meniscal root tear
Subacute or chronic grade 1 or 2 PCL rupture for which nonoperative treatment has failed
Kellgren-Lawrence grade 0-2 chondral degeneration

PCL, posterior cruciate ligament.

## Surgical Technique

In the setting of MLKI, the procedure is typically performed under general anesthesia with a regional nerve block. A thigh tourniquet is used for hemostasis. The patient is placed in the supine position on a standard operating table. The foot of the bed may be lowered, or the leg may be draped over the side of the table with use of a lateral post.

### Approach and Diagnostic Arthroscopy

Standard anteromedial and anterolateral arthroscopic portals are established. A safety incision is used at the posteromedial aspect of the proximal tibia for preparation of the PCL tibial tunnel. This incision is made along the posteromedial tibia just below the tibial flare, and blunt dissection is carried out deep to the medial gastrocnemius muscle to allow for palpation of the mamillary bodies juxtaposed to the tibial PCL insertion. This also allows protection of the posterior neurovascular structures and meniscal root insertion.

**Fig 1:**
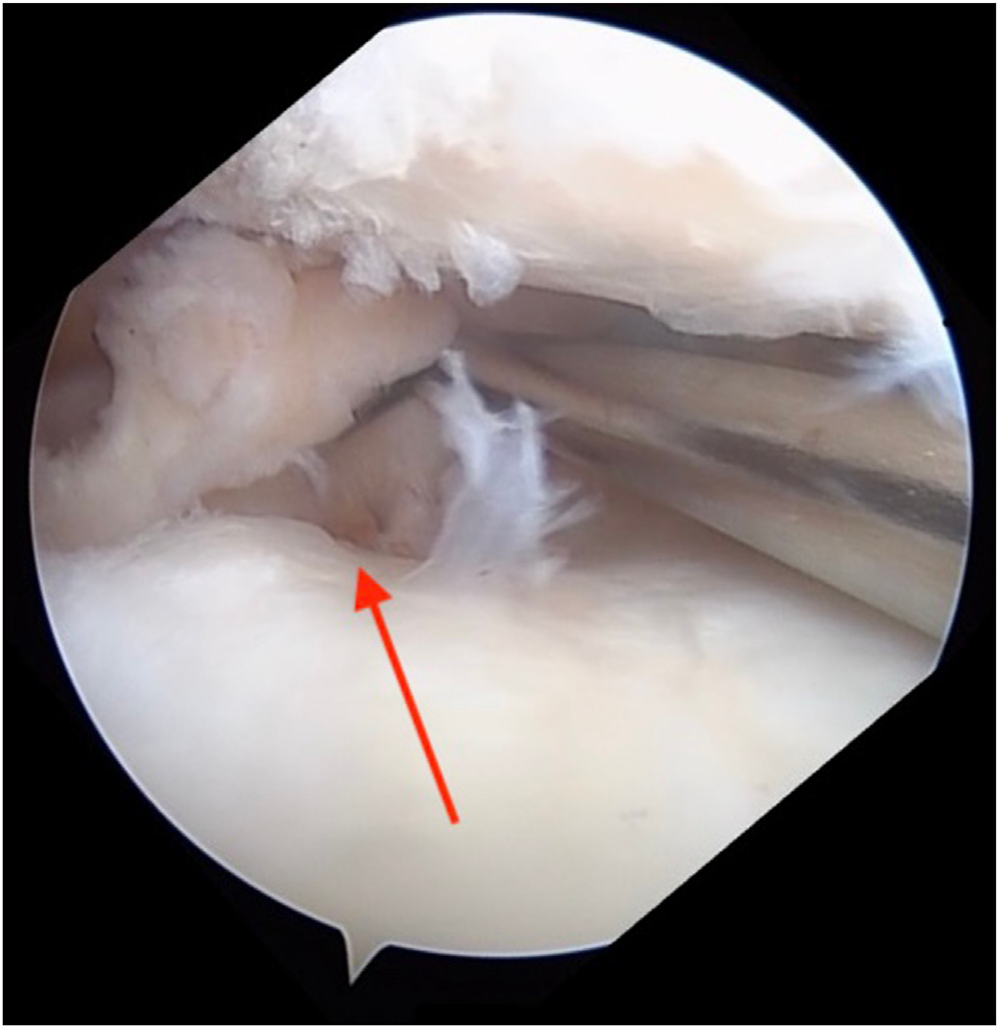
Arthroscopic image of the right knee showing the medial meniscal root tear (arrow) as it is displaced with a probe.

**Fig 2:**
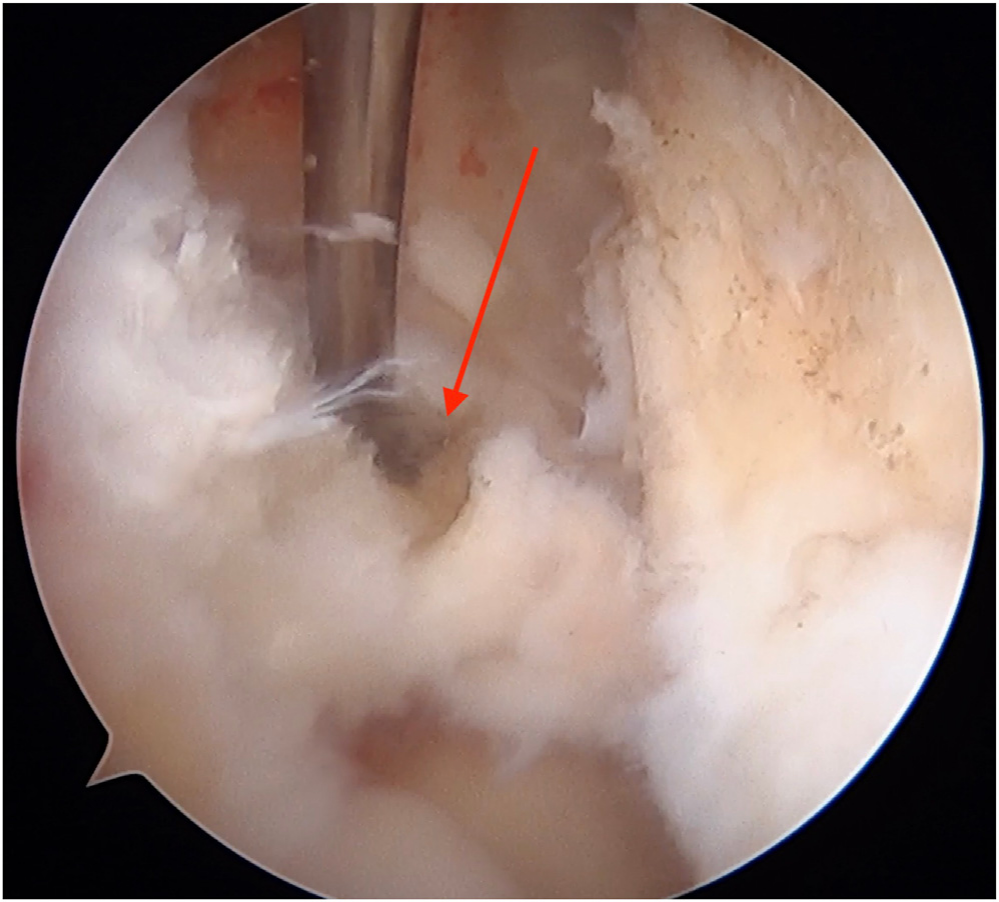
Arthroscopic image of the right knee showing preparation of the posterior cruciate ligament footprint as a rasp is used to elevate the capsule off the posterior proximal tibia down to the fovea (arrow).

Diagnostic arthroscopy is performed to confirm intra-articular pathology, debride the torn cruciate ligament(s), and identify and prepare the cruciate tunnel and meniscal root repair sites (Fig 1, Video 1). A rasp is used to prepare the PCL footprint down to the fovea by elevating the capsule off the posterior proximal tibia and to facilitate drill guide placement (Fig 2).

### Tibial Tunnels and Meniscal Root Repair

**Fig 3:**
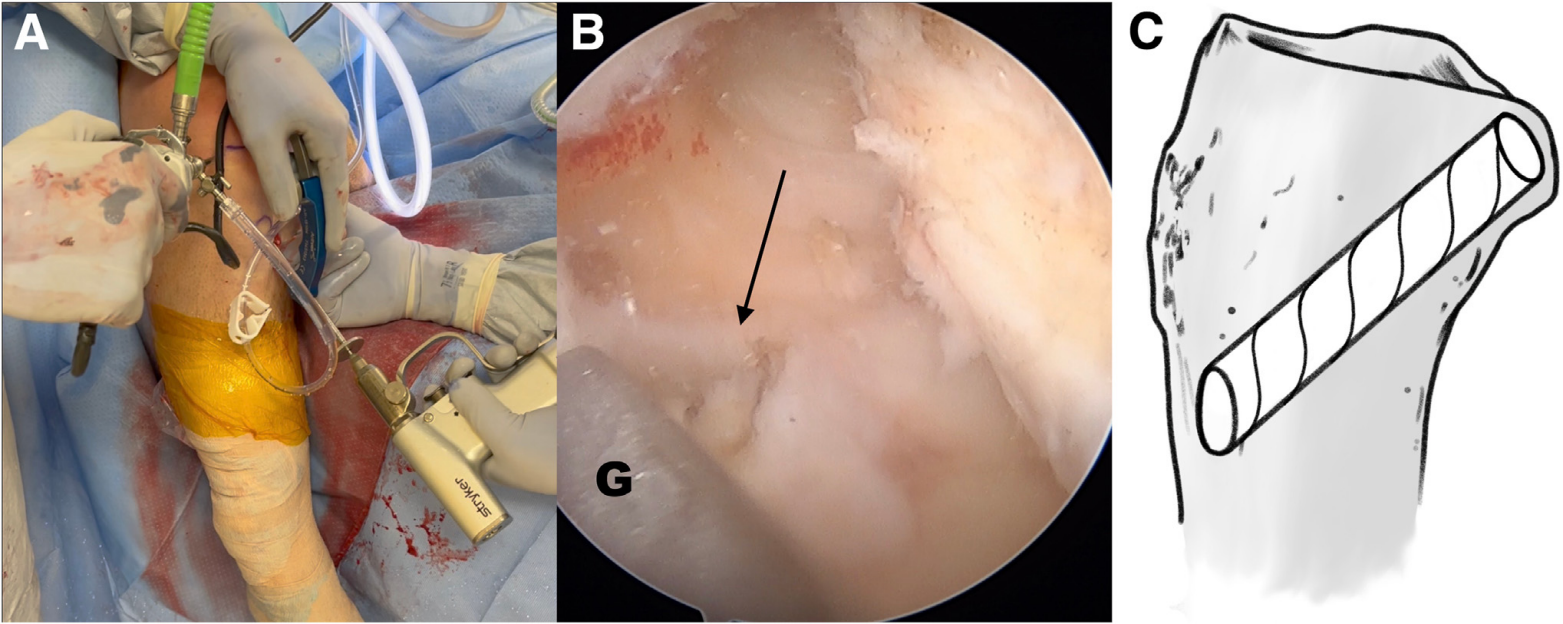
(A, B) Photograph of the anterior right knee (A) and arthroscopic view of the posterior cruciate ligament (PCL) footprint (B). The PCL tunnel is created with a 2.4-mm guide pin, which is advanced through a PCL tunnel guide (G) to the posterior cortex of the tibia. A 12-mm cannulated reamer is then advanced over the pin through the posterior cortex (arrow). (C) Sagittal view of proximal tibia with ideal PCL tunnel. (Illustration by Ryan Price, M.D.)

The PCL tibial tunnel is first prepared using the safety incision. A PCL tibia guide is placed at the appropriate anatomic footprint. A 2.4-mm guide pin is advanced to the posterior cortex. A finger is placed through the safety incision to manually protect the adjacent neurovascular structures and support the tibial guide. The 12-mm cannulated reamer is then advanced over the pin through the posterior cortex (Fig 3). Prior to breaching the posterior tibial cortex, the guide pin is removed and the blunt end is placed back in the tunnel for final drilling.

A nitinol passer is placed up the PCL tibial tunnel to pass a suture that is retrieved through the anteromedial portal. A chamfering device is then advanced using the passing suture through the anteromedial portal to chamfer the edge of the tunnel. The chamfering device is subsequently used to place 2 passing sutures through the tunnel for later graft passage. The chamfering device facilities smooth graft passage while still leaving a small capsular envelope to maintain fluid pressure.

**Fig 4:**
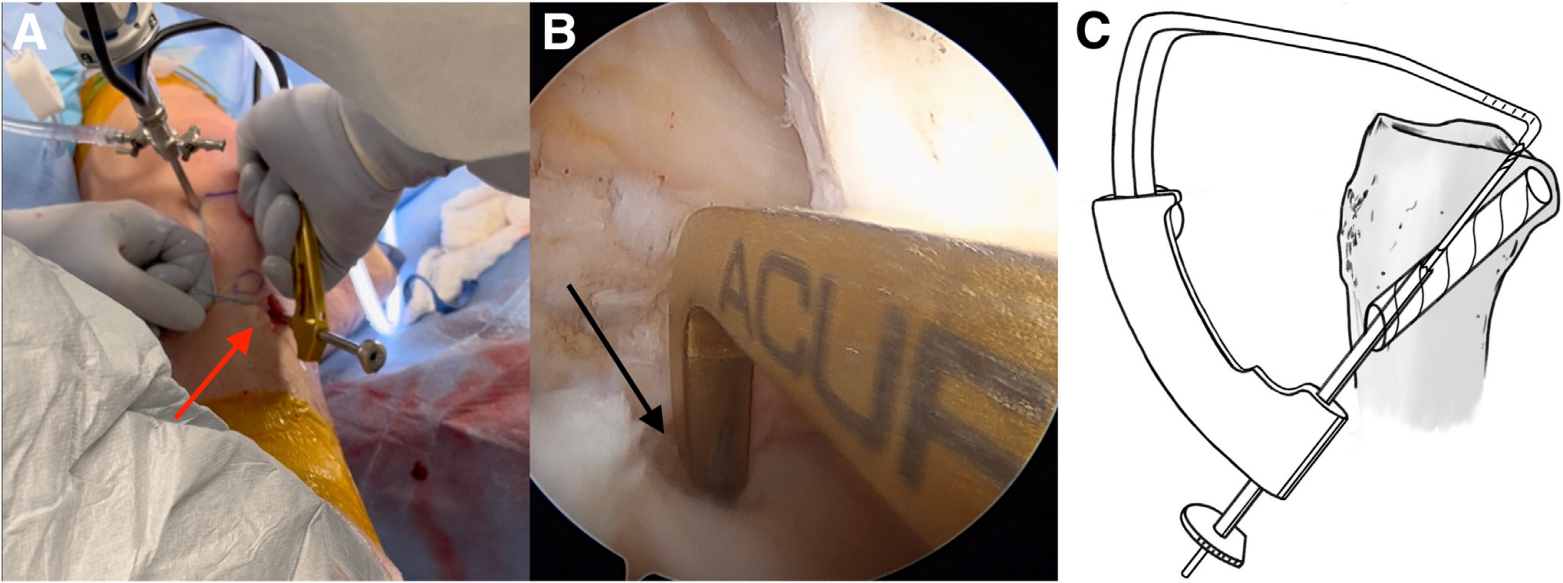
(A, B) Photograph of the anterior right knee (A) and arthroscopic view of the medial meniscal root attachment within the knee (B). The 4-mm meniscal root tunnel is created with a drill guide placed at the anatomic meniscal root footprint (black arrow), while the starting point of the guide is placed within the posterior cruciate ligament tibial tunnel using the same external aperture at the proximal anteromedial tibia (red arrow). (C) Sagittal view of proximal tibia with ideal guide placement. (Illustration by Ryan Price, M.D.)

**Fig 5:**
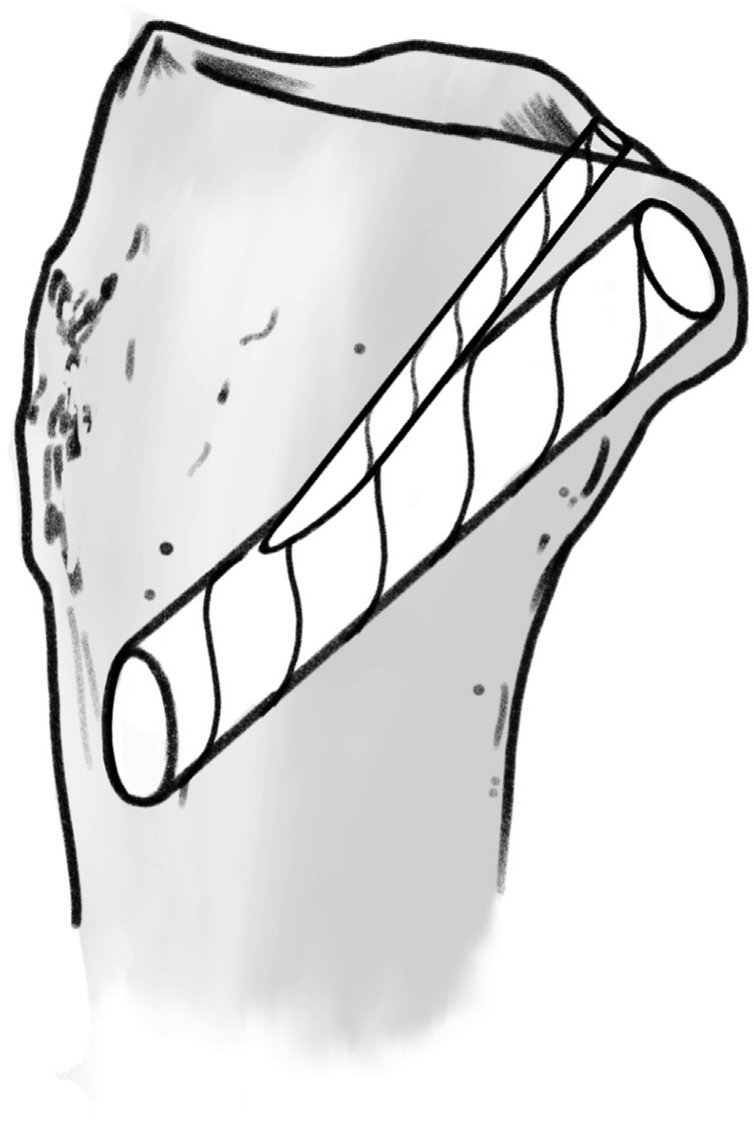
Sagittal view of the proximal tibia with the final Y-shaped convergence of the 4-mm meniscal root tunnel and 12-mm posterior cruciate ligament tunnel with a common distal tunnel. (Illustration by Ryan Price, M.D.)

**Fig 6:**
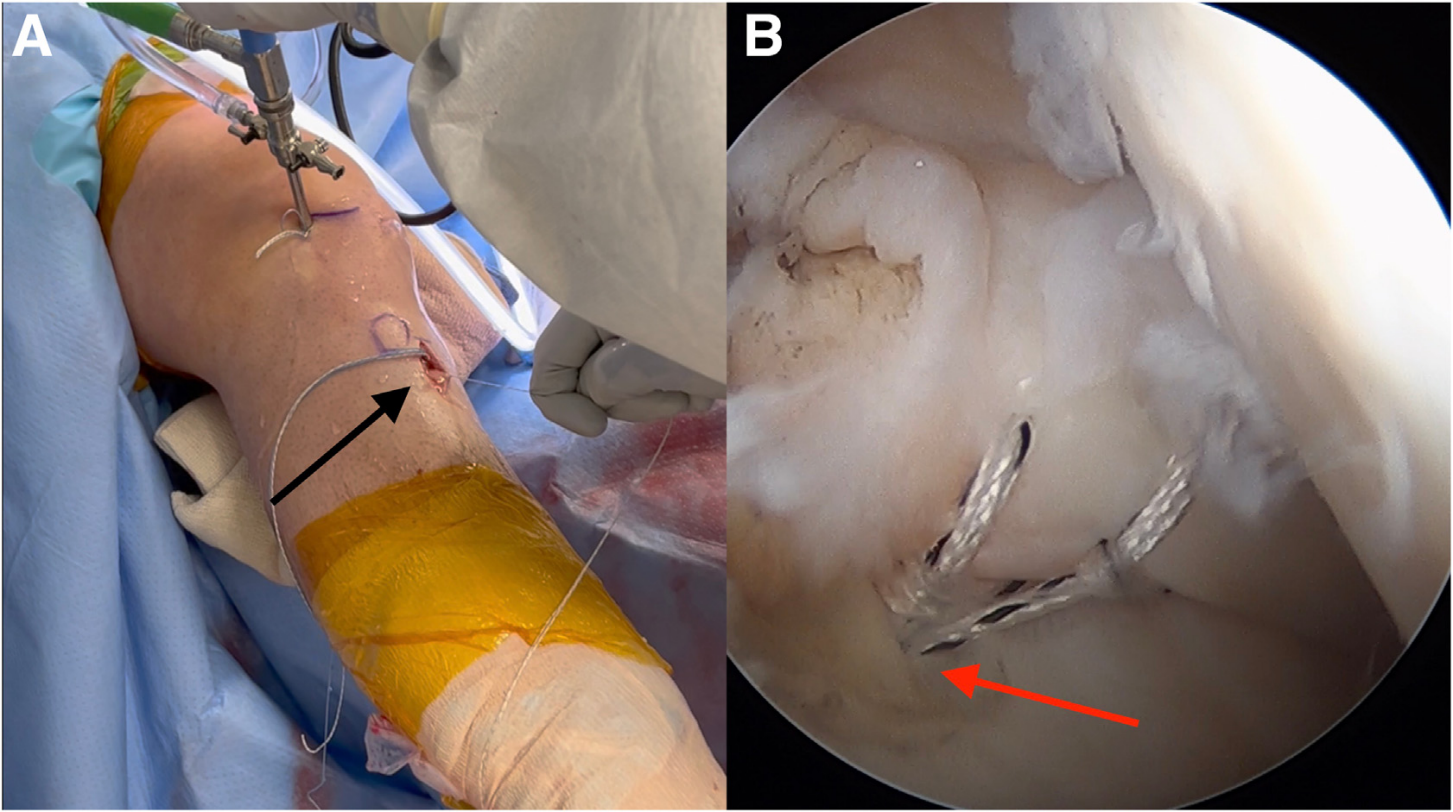
Photograph of the anterior right knee (A) and arthroscopic view of the medial meniscal root (B) as it is provisionally fixated through the root tunnel (red arrow) and the common anterior tibial aperture (black arrow).

An anterior cruciate ligament (ACL) drill guide (or meniscal root repair guide) is placed at the anatomic footprint of the medial or lateral meniscal root. The distal aspect of the guide is placed within the PCL tibial tunnel using the same external aperture at the anteromedial tibia (Fig 4). A 2.4-mm pin is advanced to the root footprint. A 4-mm cannulated drill is then used to create the root tunnel starting from within the PCL tunnel. The result is a Y-shaped tunnel with a common aperture (Fig 5) to allow for controlled collision. A No. 0 Prolene suture (Ethicon, Somerville, NJ) is then passed through the cannulated drill. A suture-passing device is used to pass 2 No. 0 FiberLink cinch sutures (Arthrex, Naples, FL) in the meniscal root. These are retrieved and then shuttled through the tibial tunnel using the No. 0 Prolene (Fig 6).

### Femoral Tunnels

**Fig 7:**
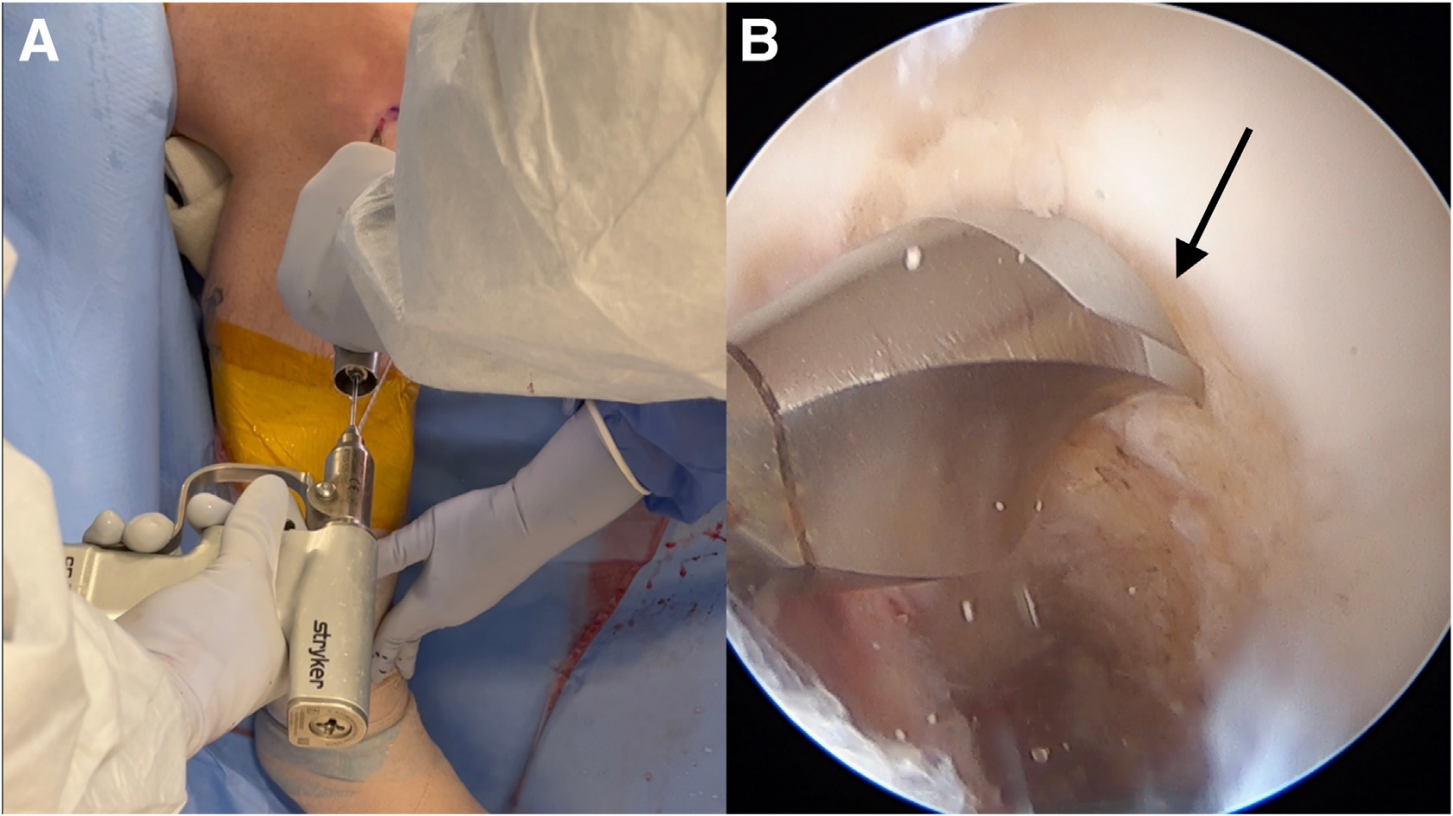
Photograph of the anterior right knee (A) and arthroscopic view of the knee with the planned trajectory of the posterior cruciate ligament anterolateral bundle tunnel (B). A 10- or 11-mm reamer is placed at the bundle femoral footprint (arrow), and a 2.4-mm guide pin is advanced through the reamer. The pin is advanced through the femur and out the distal anteromedial thigh. The reamer is then advanced over the pin, creating a socket with a depth of 20 to 25 mm.

**Fig 8:**
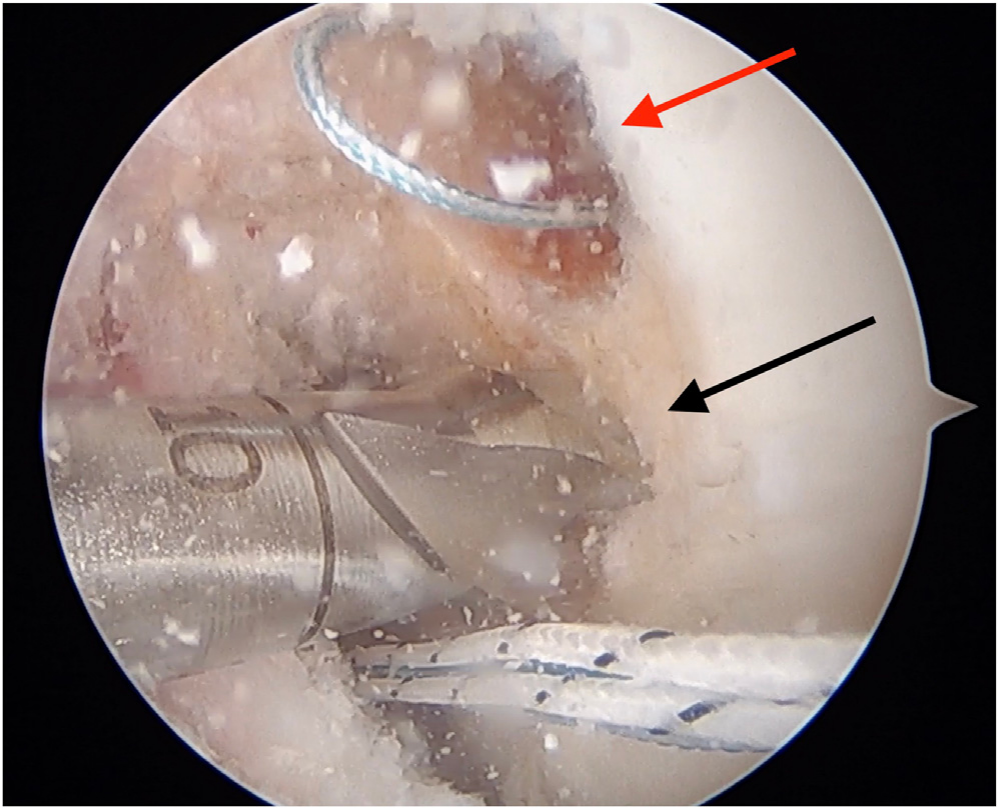
Arthroscopic image of the right knee in which the trajectory of the posterior cruciate ligament posteromedial bundle tunnel is planned and then drilled using a 7-mm reamer. The femoral footprint (black arrow) is identified at the medial wall; then, a pin is advanced within the reamer. The socket is reamed to a depth of 25 mm. The posterior cruciate ligament anterolateral bundle tunnel can be seen anterior to the reamer (red arrow).

Next, the femoral tunnels for the PCL are prepared. The anterolateral bundle tunnel is first drilled, located higher in the notch between the trochlear notch point and medial arch point (Fig 7). A 10- or 11-mm reamer is placed at the footprint to serve as a guide, and a 2.4-mm guide pin is advanced through the reamer. The pin is advanced through the femur and out the distal anteromedial thigh. The reamer is then advanced over the pin, creating a socket with a depth of 20 to 25 mm; in this case, the bone block is 23 mm, so this depth is drilled. The posteromedial bundle tunnel is prepared using a 7-mm reamer. The footprint is identified at the medial wall; then, the pin is advanced within the reamer (Fig 8). The socket is reamed to a depth of 25 mm, and a passing stitch is placed using the eyelet from the pin. A passing suture is then docked in both the anterolateral and posteromedial tunnels using the eyelet in the guide pin.

### Graft Preparation

**Fig 9:**
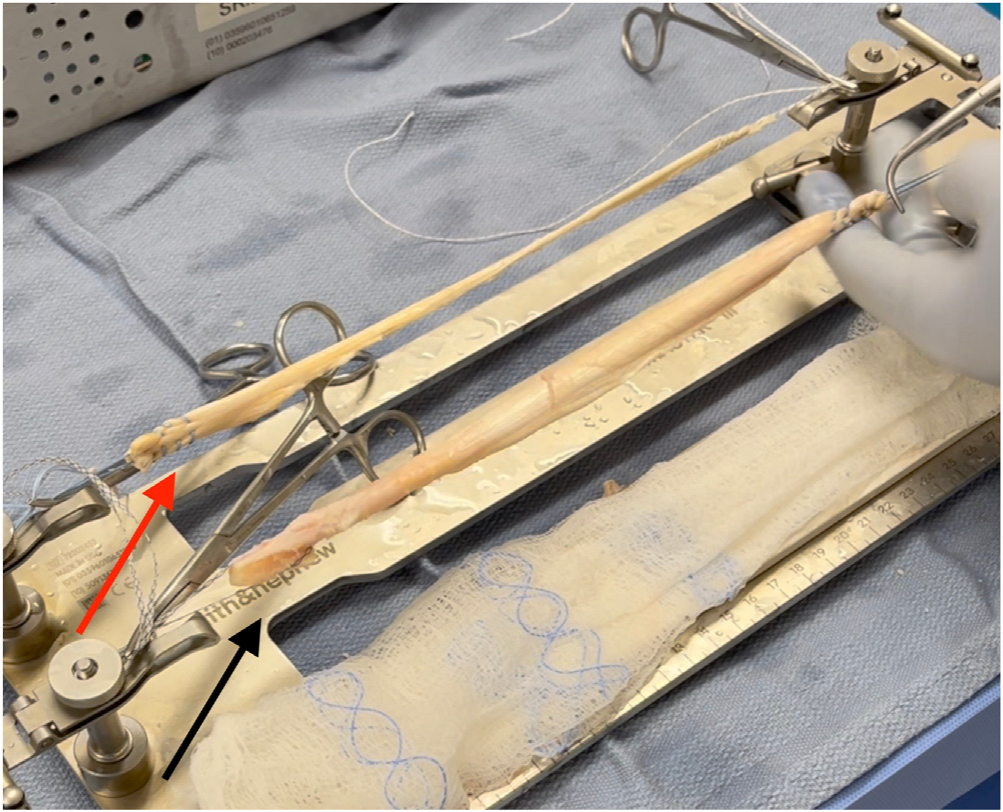
Photograph of the posterior cruciate ligament (PCL) double-bundle grafts held in tension on the back table. A tibialis anterior allograft (red arrow) is used for the posteromedial bundle, and an Achilles bone block allograft (black arrow) is used for the anterolateral bundle.

We prefer a double-bundle technique for PCL reconstruction; however, single- or double-bundle reconstruction can be used. For a double-bundle reconstruction, we use a tibialis anterior allograft for the posteromedial bundle and an Achilles bone block graft for the anterolateral bundle. The posteromedial graft (tibialis anterior) is whipstitched on the femoral and tibial ends and tapered on the tibial end. It is secured with 2 No. 2 nonabsorbable sutures. The femoral end should fit snugly in a 7-mm-diameter sizer. For the anterolateral bundle graft (Achilles bone block), the bone block is cut to a length of 23 mm, which fits snugly in a 10-mm-diameter sizer for female patients and in a 11-mm-diameter sizer for male patients. The tibial end is sutured with a No. 2 nonabsorbable suture, and a 1.6-mm drill is used to pass 2 to 3 nonabsorbable sutures through the bone block. Both grafts are placed on tension on the back table and covered with a vancomycin-wrapped Ray-Tec sponge (Johnson & Johnson, New Brunswick, NJ) (Fig 9).

### Graft Passage

**Fig 10:**
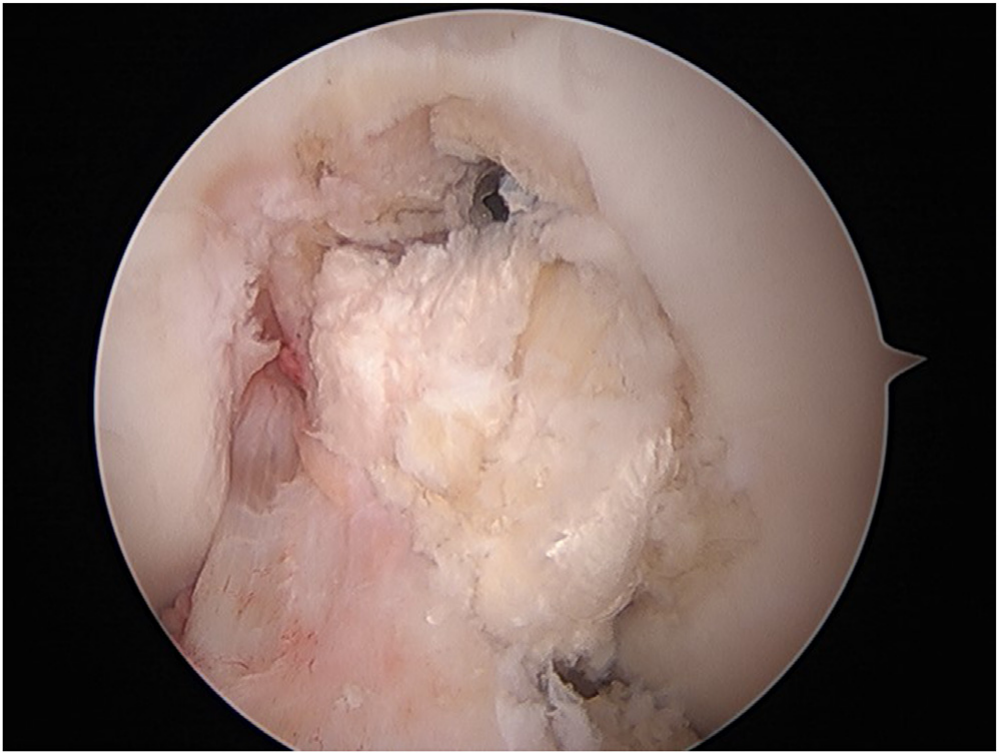
Arthroscopic image of the right knee showing the final placement of the double-bundle posterior cruciate ligament grafts in the correct anatomic orientation.

Before graft passage, the anterolateral passing suture from the tibia is pulled out through the anteromedial portal so that it is not lost during passage of the posteromedial graft. The posteromedial graft is first passed into the femur and fixed with a PEEK (polyether ether ketone) interference screw. The anterolateral bundle graft is then passed in similar fashion and fixed with a metal interference screw (Fig 10).

The meniscal root sutures are tensioned, reducing the root to the anatomic footprint with fixation to the tibia using a knotless suture anchor (4.75-mm SwiveLock; Arthrex). The PCL grafts are fixed on the tibial side. We first fix the anterolateral bundle using a 4.5-mm bicortical screw with a spiked washer. The graft is split, and the screw is inserted within the split. The graft is tensioned at 90° of knee flexion while an anterior drawer forced is applied. The posteromedial bundle is fixed using a PEEK interference screw with the graft tensioned while the knee is in full extension.

### Postoperative Rehabilitation

The patient is placed in a hinged knee brace or PCL support brace and instructed to remain non–weight bearing for 6 weeks. Knee range of motion from 0° to 90° while in bed is allowed, but the brace is locked when mobilizing. Avoidance of hamstring activation is encouraged. Aspirin, 81 mg twice a day, is prescribed for deep venous thrombosis prophylaxis for 2 weeks postoperatively. Physical therapy begins at 2 weeks postoperatively as tolerated and should continue as long as needed to regain full strength and range of motion.

## Discussion

Unintended tunnel collision can lead to injury to grafts or fixation devices. Proximity of tunnels also raises concerns about osteolysis and loss of fixation. The placement of meniscal root tunnels can significantly increase the risk of tunnel collision in MLKI surgery.

Multiple surgical techniques have been described to avoid unintended tunnel convergence, particularly with concomitant ACL reconstruction and meniscal root repair. These include using a lateral tibial tunnel or even using the same tibial tunnel aperture in a similar technique to what we describe.^7^^,^^8^ Gursoy et al.^6^ used 3-dimensional modeling to show a significant risk of tunnel convergence between the ACL and lateral meniscal root tunnels when the root tunnel’s entry was created proximal to the ACL tunnel’s entry. They recommended reorienting the root repair tunnel parallel to the ACL tunnel in the sagittal plane such that their aperture is distal along the anteromedial tibia. However, they found that the risk of PCL convergence increased depending on the type of root tunnel construction and cautioned against a double-tunnel root repair technique.^6^ The present technique addresses this issue with PCL reconstruction by intentionally converging these tunnels (i.e., “controlled collision”) and, in so doing, reduces the tunnel burden in the proximal tibia. In our experience, this has helped reduce tunnel traffic without compromising the integrity of the grafts or fixation. Pearls and pitfalls are described in Table 2. Advantages and disadvantages are described in Table 3.

**Table 2:** Pearls and Pitfalls

Pearls	Pitfalls
A safety incision should be used at the posteromedial aspect of the proximal tibia for preparation of the PCL tibial tunnel.	Nonanatomic PCL tibial tunnel placement is possible, and there is a risk of damage to the posterior neurovascular structures or meniscal root insertions.
A rasp should be used to prepare the PCL footprint down to the fovea to facilitate drill guide placement.	The surgeon may encounter inappropriate PCL tunnel placement and difficulty with graft passage due to excessive soft tissue.
During PCL tunnel drilling, the surgeon should remove the guide pin and place the blunt end back in the tunnel for final drilling.	Accepting nonanatomic posterior root placement should be avoided.
The surgeon should ensure the correct order of graft tensioning on the tibial side. The anterolateral bundle graft is first tensioned at 90° of knee flexion while an anterior drawer forced is applied. The posteromedial bundle is fixed with the graft tensioned while the knee is in full extension.	The surgeon may encounter inappropriate graft tensioning with either hyperlaxity or inappropriate stiffness, or ultimate graft failure may occur.

PCL, posterior cruciate ligament.

**Table 3:** Advantages and Disadvantages

Advantages
Avoidance of unintended tunnel collision and potential injury to graft or fixation devices
Avoidance of tunnel osteolysis and loss of fixation owing to proximity of tunnels
Available technique for either medial or lateral meniscal root tears
Can be used with variety of PCL reconstruction techniques or graft fixation methods
Disadvantages
Higher complexity of suture management through single tunnel aperture

PCL, posterior cruciate ligament.

The primary limitation of this technique is an absence of long-term data assessing what impact this may have on graft strength or fixation. Thus far, we have not experienced any unusual complications. The use of any new technique has a learning curve, although the theoretical risk of this technique is low. Suture management is perhaps slightly more complex, and ensuring the correct order of tensioning is of greater importance. In the setting of concomitant injury to the medial or lateral posterior meniscal root and PCL, this technique eliminates inadvertent tunnel collision risk between the meniscal root and the PCL tunnels, reduces tunnel volume in the limited proximal tibial bone mass, and reduces tunnel traffic at the proximal anteromedial tibia without compromising the integrity of the grafts or fixation.

## Disclosures

All authors (N.N., R.P., K.Y., C.C., J.E., D.R., C.S., G.T.) declare that they have no known competing financial interests or personal relationships that could have appeared to influence the work reported in this paper.

## Supplementary Data

Video 1 A preoperative posterior drawer test confirms right knee posterior cruciate ligament (PCL) laxity. Diagnostic arthroscopy is performed to confirm intra-articular pathology, debride the torn PCL, and identify and prepare the cruciate tunnel and meniscal root repair sites. A rasp is used to prepare the PCL footprint. A PCL tibial guide is placed, and a 12-mm cannulated reamer is advanced over a guide pin. A nitinol passer is advanced through the PCL tibial tunnel, followed by a passing suture and then a chamfering device. An anterior cruciate ligament (ACL) drill guide is placed at the anatomic footprint of the medial meniscal root, with the distal aspect placed within the PCL tibial tunnel using the same external aperture. A 2.4-mm pin, followed by a 4-mm cannulated drill, is used to create the root tunnel starting from within the PCL tunnel. Two cinch sutures are placed in the meniscal root, which are shuttled through the tibial tunnel. The femoral tunnel for the PCL anterolateral bundle is prepared with an 11-mm reamer over a 2.4-mm guide pin to create a socket depth of 23 mm. The femoral tunnel for the PCL posteromedial bundle is prepared using a 7-mm reamer to a depth of 25 mm. A tibialis anterior allograft is used for the PCL posteromedial bundle, and an Achilles bone block graft is used for the anterolateral bundle. The posteromedial graft is passed into the femur and then into the tibia. The femoral side of the graft is subsequently fixed with a PEEK interference screw. The anterolateral bundle graft is passed in similar fashion and fixed with a metal interference screw. The meniscal root sutures are then tensioned and fixed to the tibia using a knotless suture anchor. The PCL anterolateral bundle is then fixed on the tibial side while tensioned at 90° of knee flexion while an anterior drawer forced is applied. The posteromedial bundle is fixed with the graft tensioned while the knee is in full extension.
